# Health assessment of important tributaries of Three Georges Reservoir based on the benthic index of biotic integrity

**DOI:** 10.1038/s41598-020-75746-7

**Published:** 2020-10-30

**Authors:** Zongfeng Li, Bo Zeng

**Affiliations:** Key Laboratory of Eco-environments in Three Gorges Reservoir Region (Ministry of Education), School of Life Sciences, Southwest China University, No. 2 Tiansheng Road, Beibei District, Chongqing, 400715 China

**Keywords:** Ecology, Ecology, Hydrology

## Abstract

China’s Three Gorges Reservoir (TGR) is the largest water conservancy project in the world, and plays a significant role in flood control and water transport. To study the health status of the tributaries of TGR, we set up 175 sampling sites, including 15 reference sites and 160 impaired sites on 36 important tributaries of TGR, and collected zoobenthos at these sampling sites. We selected 26 candidate metrics, analyzed them in terms of the distribution range, discriminant ability and correlation. Eight core metrics (i.e., total taxa, ephemerida + trichoptera + plecoptera taxa, diptera taxa, ephemerida%, trichoptera%, Shannon–Wiener diversity index, dominant species% and filter%) were identified, and then the benthic indexes of biological integrity (B-IBI) was established. The B-IBI was then used to evaluate the ecological status of 36 tributaries of TGR. Among all the sampling sites, “excellent”, “good”, “fair”, “poor” and “very poor” accounted for 5.14%, 13.14%, 28.00%, 44.57%, and 9.14%, respectively. Among all streams and rivers, “excellent”, “good” “fair”, “poor” and “very poor” accounted for 5.56%, 41.67%, 50.00%, 2.78%, and 0%, respectively, showing a general good condition for all tributaries. There was a significant difference in health status between tributaries in the TGR dam and those in its upper reaches. The B-IBI established in this study can capture the health status of 36 important tributaries of TGR. This study does not only enrich the practice of health assessment using B-IBI, but also provides some reference for the evaluation of similar rivers across the world.

## Introduction

Application of index of biotic integrity (IBI) can evaluate environment by going beyond the constraints of either physical–chemical indexes or merely biotic community, and extending the evaluation to include the impact of the comprehensive habitat, community structure, community function, family, genus, species and other biological taxa on the overall ecosystem; therefore, it has been an important method for evaluating and managing river ecosystem^1–4^.

Karr was the first to construct the index of biological integrity (IBI) for use with fish to evaluate river health, which was gradually applied to aquatic biological groups such as macrobenthos, algae and plankton. At present, the aquatic ecological health evaluation technology based on fish and benthos is the most popular and sophisticated, and aquatic ecological health evaluation based on macrobenthos has been widely applied to the research on and management of aquatic ecology and water environment. Wright et al.^5^ used macro-invertebrate species to study the health status of 41 river systems in Britain and revealed the correlation between macro-invertebrate species and types of rivers. Barbour et al.^6^ used benthic assemblages to classify river conditions in Florida, USA, and screened out 8 biological metrics for classification. Dauer et al.^7^ studied associations between macrobentho community, dissolved oxygen in water and anthropogenic activities in the whole basin for Chesapeake Bay, USA, showing that dissolved oxygen and anthropogenic activities greatly impacted macrobenthos community. Maxted et al.^8^ studied the health status of 106 streams along the Atlantic coast of the United States using benthic macroinvertebrates. They established the Coastal Plain Macroinvertebrate Index (CPMI), and accurately described the habitat disturbance and water quality impairment. Karr^9^ used the benthic index of biotic integrity (B-IBI) to assess stream health in Puget Sound, Washington State, United States, and applied it to the management of urban rivers. Hering et al.^10^ established a framework for streams in Europe based on benthic macroinvertebrates, including initial assessment methods for 28 types of streams in Europe and biological monitoring tools applicable to European rivers. Bilkovic et al.^11^ studied the relationship between macrobenthic community index and shoreline alteration and watershed land use in the coastal estuaries of Chesapeake Bay, USA, and found that macrobenthic community indices were greatly impacted by nutrient influxes and watershed land use. Gabriels et al.^12^ developed and tested the river and lake evaluation index, i.e., Multimetric Macroinvertebrate Index Flanders (MMIF), based on macroinvertebrate samples, which can be used for an overall assessment of ecological deterioration caused by any kind of stressor. Zhang et al.^13^ studied the impact of land use differences on benthic macroinvertebrate communities in Xitiaoxi River basin in China, and discovered that land use differences would result in a significant difference in the composition of macroinvertebrate community. Based on the B-IBI index selection principle, Wang et al.^14^ identified two biological metrics, i.e., macroinvertebrate species richness and number of ephemerida + trichoptera + plecoptera (EPT) taxa, from 27 biological metrics in their study of the Xiangxi River Basin in China, and assessed the impact of river dam operation on macroinvertebrates using these two metrics. Cai et al.^15^ established a B-IBI for Taihu Lake in China, and used it to evaluate the ecosystem health of Taihu Lake, pointing out that continuous data observation can improve the accuracy of the B-IBI evaluation. Lau et al.^16^ conducted an in-depth study in the Ohio River basin and perfected the benthic macroinvertebrate multimetric indices (MMIs), making it convenient for the US Environmental Protection Agency to identify impaired streams and sources of those impairments without being constrained by state-by-state developed assessment methods.. Jose et al.^17^ studied the B-IBI, an environmental evaluation index developed for Chesapeake Bay, revealing that improvement can be made by recalibrating the existing index threshold or selecting a new index. Cui et al.^18^ established an adapted B-IBI and successfully evaluated the ecosystem health assessment of Zhanghe River basin. Lu et al.^19^ evaluated the floodplain wetland using aquatic invertebrates in the Wusuli River in northeast China, and concluded that levee construction has a consistent negative impact on the conditions of the floodplain wetland. With the advancement of science, there will be more and more evaluation studies. Moreover, the methods for river health assessment based on the benthic index are becoming more and more sophisticated.

The Three Gorges Reservoir in China is an artificial lake formed due to the impoundment after the completion of the TGP. It has a total area of 1084 km^2^ and has 36 important tributaries, covering Hubei Province and Chongqing City. After the completion of the Three Gorges Reservoir (TGR), the water level gradually rises to 175 m and maintain at a high level as much as possible, with a total reservoir capacity of 39.3 billion m^3^. Anthropogenic activities exert a big impact on the health of river ecosystems, despite the large water area of TGR, a quite few feeding tributaries, and a vast basin area. The tributaries flowing into the reservoir play a very important role in protecting and maintaining the normal function of the reservoir’s aquatic ecosystem. Chi et al.^20^ established a multi-metric index based on macroinvertebrates (SXMMI) and a corresponding rating criterion, and set up a sampling site on each tributary to evaluate the ecosystem health of some tributaries of the TGR. Zhang et al.^21^ analyzed the impact of land use on the water quality of tributaries of the TGR in China based on seasonal and spatial dimensions. Ma et al.^22^ created an export coefficient model (ECM) to evaluate the impact of nitrogen and phosphorus from agricultural nonpoint source on water quality in the Three Gorges Reservoir Area. Although these studies on the health status of tributaries of the TGR can reveal and shed light on scientific phenomena, there are still some deficiencies, such as not covering all important tributaries, monotonous evaluation index, few monitoring sample sites or difficulty in reaching a comprehensive assessment due to cross-watershed investigation involving multiple variables.

Therefore, in this study, we proposed a method for assessing the health of tributaries of TGR based on the benthic index of biotic integrity (B-IBI) to solve the comprehensive assessment of ecological status of cross-watershed rivers. In the meantime, this study may also provide practical reference for health assessment of similar rivers in the world.

## Results

### IBI establishment

#### Analysis of distribution range of candidate metrics

Verification of the distribution range of all candidate metrics (Table 1) revealed that as standard deviation of Pteroptera taxa (M5), Crustacean + Mollusca% (M10), Pteroptera% (M12), Chironomidea% (M15), Oligochaeta% (M16), Shredders% (M22) and Predators% (M26) were all greater than the mean or contained too many zero values, these metrics were deleted. The remaining 19 metrics were suitable for further screening as biological evaluation metrics.

**Table 1:** Distribution of candidate metricvalues in the reference sites.

Metric serial no	Mean	SD	Minimum value	Maximum value	25% quantile	Median	75% quantile
M1	27.93	7.81	13.00	45.00	22.00	28.00	30.00
M2	12.80	4.74	6.00	24.00	10.00	11.00	17.00
M3	4.73	3.10	0.00	10.00	2.00	4.00	7.00
M4	9.07	3.33	5.00	18.00	7.00	8.00	11.00
M5	0.47	0.99	0.00	3.00	0.00	0.00	0.00
M6	3.20	1.66	1.00	6.00	2.00	3.00	5.00
M7	6.20	3.84	1.00	14.00	4.00	6.00	8.00
M8	4.40	3.36	0.00	12.00	2.00	3.00	7.00
M9	0.65	0.28	0.11	0.96	0.52	0.70	0.87
M10	0.15	0.18	0.00	0.52	0.01	0.04	0.27
M11	0.42	0.26	0.04	0.86	0.13	0.46	0.63
M12	0.00	0.01	0.00	0.04	0.00	0.00	0.00
M13	0.21	0.17	0.02	0.47	0.04	0.20	0.38
M14	0.14	0.12	0.00	0.38	0.04	0.11	0.17
M15	0.10	0.12	0.00	0.33	0.01	0.04	0.17
M16	0.00	0.00	0.00	0.01	0.00	0.00	0.00
M17	3.13	0.72	1.49	3.94	2.62	3.23	3.69
M18	9.53	3.81	5.00	17.00	7.00	8.00	10.00
M19	2.07	1.10	0.00	4.00	2.00	2.00	3.00
M20	0.31	0.12	0.17	0.59	0.24	0.27	0.32
M21	0.61	0.13	0.42	0.85	0.48	0.63	0.73
M22	0.01	0.03	0.00	0.08	0.00	0.00	0.00
M23	0.51	0.15	0.32	0.82	0.33	0.53	0.63
M24	0.28	0.15	0.07	0.56	0.18	0.26	0.38
M25	0.14	0.10	0.03	0.32	0.06	0.12	0.25
M26	0.05	0.06	0.00	0.24	0.02	0.03	0.05

#### Analysis of discriminant range of candidate metrics

**Figure 1:**
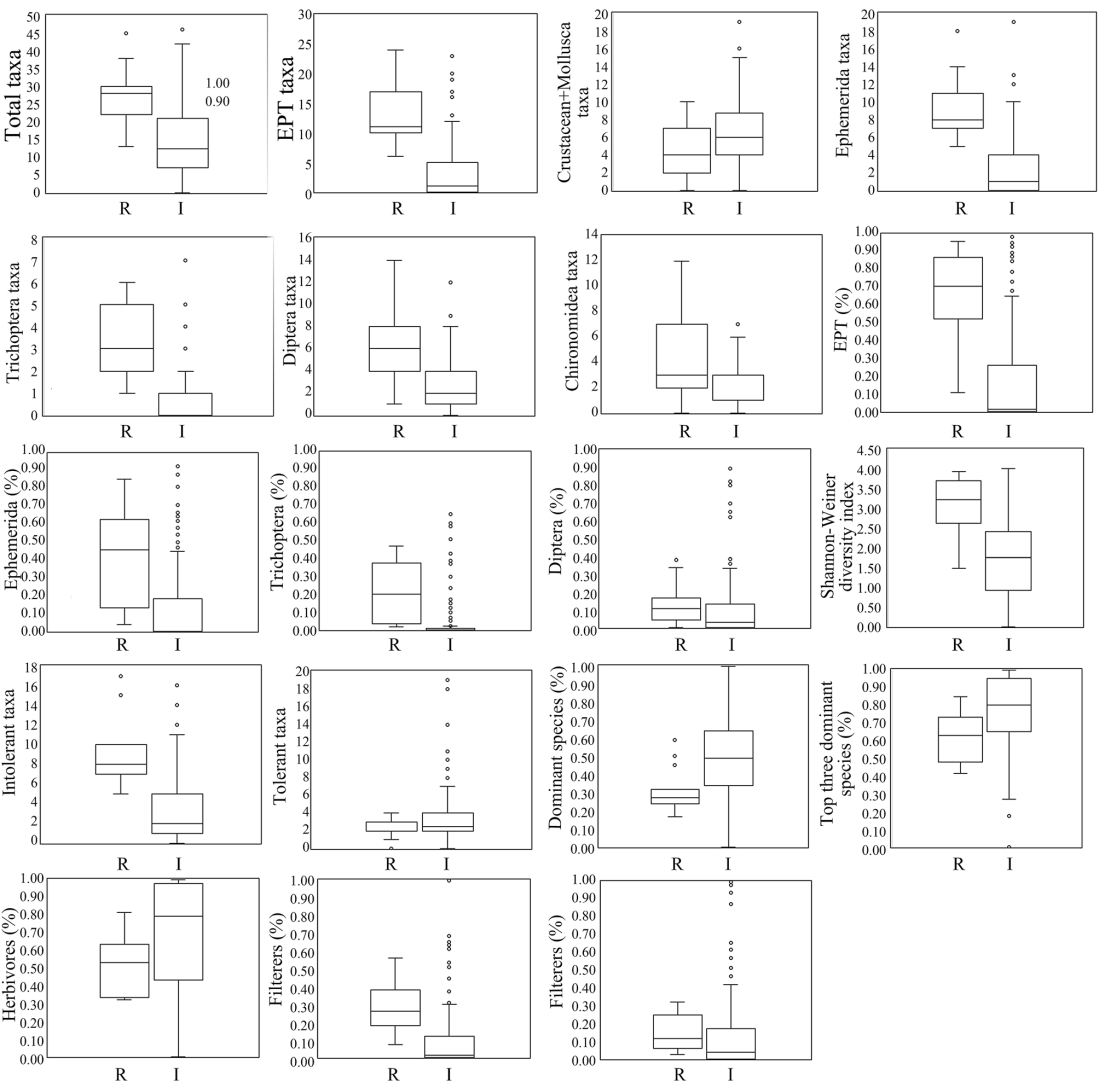
Box-plot of candidate metrics of reference sites and impaired sites. *R* reference sites, *I* impaired sites.

Discriminant analysis of the candidate metrics showed that (Crustacean + Mollusca) taxa, chironomidea taxa, diptera%, tolerance species (PTV ≥ 7), herbivore%, scrapers% were largely overlapping between reference sites and impaired sites (IQ < 2), thereby unsuitable for biological assessment (Table 2, Fig. 1). Thirteen candidate metrics, namely, total taxa, EPT taxa, ephemerida taxa, trichoptera taxa, diptera taxa, EPT%, ephemerida%, trichoptera%, Shannon–Wiener diversity index, intolerant taxa (PTV ≤ 3), dominant species%, top three dominant species% and filters%, exhibited a slight overlap (IQ ≥ 2) between the reference sites and impaired sites. Therefore, they were suitable to be used for further analysis.

**Table 2:** Degrees of overlapping between the reference sites and the impaired sites in terms of the candidate metrics.

Serial no	Biological parameter	IQ value	Serial no	Biological parameter	IQ value
M1	Total taxa	3	M13	Trichoptera (%)	3
M2	EPT taxa	3	M14	Diptera (%)	1
M3	Crustacean + Mollusca taxa	0	M17	Shannon–Weiner diversity index	3
M4	Ephemerida taxa	3	M18	Intolerant taxa (PTV ≤ 3)	3
M6	Trichoptera taxa	3	M19	Tolerant taxa (PTV ≥ 7)	0
M7	Diptera taxa	2	M20	Dominant species (%)	3
M8	Chironomidea taxa	1	M21	Top three dominant species (%)	2
M9	EPT (%)	3	M23	Herbivores (%)	1
M11	Ephemerida (%)	3	M24	Filterers (%)	3

#### Correlation analysis of candidate metrics

Taken together the correlation analysis results between candidate metrics (Table 3) and the biological significance of candidate metrics, the screening results of candidate metrics were as follows: ephemerida taxa (M4) and intolerant taxa (M18) were both significantly correlated with the total taxa (M1). As the total taxa could more fully reflect the environmental characteristics of the community, ephemerida taxa and intolerant taxa were eliminated. EPT taxa was significantly correlated with ephemerida taxa, trichoptera taxa, and EPT%. Since EPT taxa included ephemerida taxa and trichoptera taxa and was closely related to EPT%, therefore, EPT taxa was retained, while ephemerida taxa, trichoptera taxa, and EPT% were eliminated. Since the dominant species% contained the information of the top three dominant species%, the dominant species% was retained, while the top three dominant species% was deleted.

**Table 3:** Correlation analysis results for 13 candidate metrics.

	M1	M2	M4	M6	M7	M9	M11	M13	M17	M18	M20	M21	M24
M1	1.00												
M2	0.73	1.00											
M4	0.76	0.96	1.00										
M6	0.58	0.86	0.78	1.00									
M7	0.69	0.57	0.58	0.47	1.00								
M9	0.50	0.80	0.77	0.77	0.39	1.00							
M11	0.48	0.71	0.70	0.59	0.35	0.89	1.00						
M13	0.30	0.55	0.50	0.68	0.26	0.68	0.30	1.00					
M17	0.39	0.52	0.48	0.48	0.44	0.51	0.43	0.39	1.00				
M18	0.76	0.86	0.88	0.70	0.48	0.69	0.62	0.47	0.46	1.00			
M20	− 0.40	− 0.43	− 0.42	− 0.37	− 0.37	− 0.43	− 0.39	− 0.29	− 0.71	− 0.39	1.00		
M21	− 0.43	− 0.39	− 0.39	− 0.33	− 0.36	− 0.37	− 0.33	− 0.25	− 0.54	− 0.36	0.84	1.00	
M24	0.39	0.38	0.40	0.36	0.27	0.32	0.15	0.45	0.20	0.40	− 0.25	− 0.26	1.00

Through the above analysis, core metrics of B-IBI used for assessment of important tributaries of TGR included the total taxa, EPT taxa, diptera taxa, ephemeropter%, trichoptera%, Shannon–Wiener diversity index, dominant species% and filters%.

### Integrity assessment of important tributaries of TGR

#### B-IBI establishment

Using the method described in literature^23^, the values of biological metrics were standardized, the metrics were unified, and then standardized formulae for B-IBI assessment metrics of important tributaries of TGR were established (Table 4). We combined these metrics to obtain B-IBI scores of various sampling sites. The theoretical range of the index is 0–8. A smaller value means a worse health condition for streams and rivers, and vice versa. The total taxa, EPT taxa, diptera taxa, ephemerida% trichoptera% and Shannon–Wiener diversity index were the metrics that decreased with an increase in disturbance, while the dominant species% and filters% were the metrics that augmented with an increase in disturbance.

**Table 4:** Core metrics and calculation formulas.

Biological metric	Computational formula
Total taxa	Total taxa/32
EPT taxa	EPT taxa/16
Diptera taxa	Diptera taxa/7.30
Ephemerida%	Ephemerida%/0.68
Trichoptera%	Trichoptera%/0.41
Shannon diversity index	Shannon diversity index/3.69
Dominant species%	(1-dominant species%)/0.80
Filters%	(1-filter%)/1.00

#### B-IBI assessment results

1. B-IBI assessment results of the sampling sites. For each sampling site, the standardized values of the core metrics were summed up to obtain the B-IBI value. The 95% quantile of the actual distribution range of B-IBI values at all sampling sites were set as the “health” standard for sampling sites (6.54), divided into 5 grades (Table 5): excellent (M ≥ 6.54), good (4.91 ≤ M < 6.54), fair (3.27 ≤ M < 4.91), poor (1.64 ≤ M < 3.27), and very poor (< 1.64).

**Table 5:** B-IBI health assessment grade of the sampling sites.

Health grade	Excellent (M ≥ 6.54)	Good (4.91 ≤ M < 6.54)	Fair (3.27 ≤ M < 4.91)	Poor (1.64 ≤ M < 3.27)	Very poor (M < 1.64)
Number	9	23	49	78	16
Percentage	5.14	13.14	28.00	44.57	9.14

The health assessment results of the sampling sites show that there were 9 “excellent” sampling sites, accounting for only 5.14% of the total sampling sites, located in Guandu River (3), Daxi-F River (2), Caotang River (1), Changtang River (1), Xiao River (1), and Zhuxi River (1), respectively; 23 “good” sampling sites, accounting for only 13.14% of the total; 49 “fair” sampling sites, accounting for 28.00% of the total; 78 “poor” sampling sites, accounting for up to 44.57% of the total. There were 16 “very poor” sampling sites, accounting for 9.14% of the total, located in Qinggan River (1), Baolong River (1), Daning River (1), Meixi River (1), Changtan River (1), Modaoxi River (1), Xiao River (2), Rangdu River (1), Huangjin River (1), Dongxi River (2), Wu River (1), Taohua River (1), Jialing River (1), and Yipin River (1), respectively.

(2) (2) B-IBI assessment results of the tributaries. The average of the B-IBI values of all sampling sites of each tributary was calculated to obtain the B-IBI value of the tributary. The 95% quantile of the actual distribution range of B-IBI values of all tributaries were set as the “health” standard (4.55) for sampling sites, divided into 5 grades (Table 6): excellent (M ≥ 4.55), good (3.41 ≤ M < 4.55), fair (2.28 ≤ M < 3.41), poor (1.14 ≤ M < 2.28), and very poor(< 1.14).

**Table 6:** B-IBI health assessment grade of sampling sites.

Health grade	Excellent (M > 4.55)	Good (3.41 ≤ M < 4.55)	Fair (2.28 ≤ M < 3.41)	Poor (1.14 ≤ M < 2.28)	Very poor (M < 1.14)
Number	2	15	18	1	0
Percentage	5.56	41.67	50.00	2.78	0.00

The tributary health assessment results show that 2 rivers, namely Guandu River (R5) and Daxi-F River (R7), were of an “excellent” status, accounting for only 5.56% of the total; 15 rivers were of a “good” status, accounting for 41.67%; 18 rivers were of a “fair” status, accounting for 50%; Taohua River (R27) was the only one with a “poor” status, accounting for 2.78%; the number of “very poor” river was zero.

**Figure 2:**
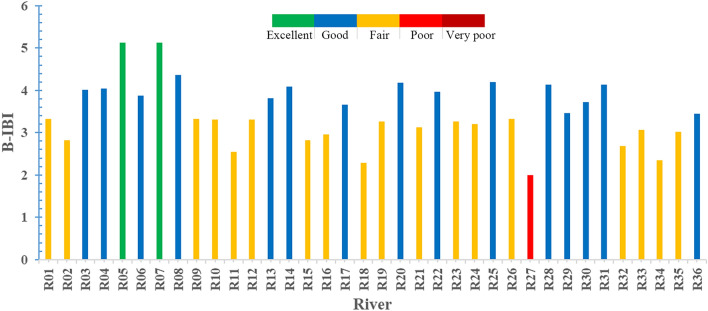
Schematic diagram of health assessment results for 36 important tributaries of TGR.

As shown in Fig. 2, there is a big difference in health status of 36 important tributaries of TGR. R05 (Guandu River) and R07 (Daxi-F River) have the best condition, namely an “excellent” status; R27 (Taohua River) has a “poor” status and is of the worst status by comparison. Comparing the tributaries in the TGR dam and in its upper reaches, the health status exhibits an overall deteriorating trend. R03–R08 are areas exhibiting a continuous “excellent” or “good” status; R28–R31 are areas exhibiting a continuous “good” status; and R09–R12 and R32–R35 are areas exhibiting a continuous “fair” status, respectively.

## Discussion

In this study, we established a river health assessment method based on B-IBI, and rationally assessed the health status of important tributaries of TGR. We set up 15 reference sites and 160 impaired sites in 36 tributaries of TGR, and selected eight biological metrics, including total taxa, EPT taxa, diptera taxa, ephemerida%, trichoptera%, Shannon–Wiener diversity index, dominant species% and filters%. We constructed a river health assessment method based on B-IBI, which was used to assess the health status of 36 important tributaries of TGR. 8 biological metrics finally selected in this study were all commonly used for assessing the ecological condition of streams and rivers^12,13,18,19,24,25^, and the tributary health status evaluation results based on B-IBI were consistent with our expectations.

The number of “very poor” and “poor” sites accounted for 53.71% of the total sampling sites, while the “excellent” and “good” sites accounted for only 18.28%. On the whole, the health status of the sampling sites on the important tributaries of TGR was in a bad shape. Sampling sites with an “excellent” and “good” status were all distributed in sparsely populated areas with a good vegetation coverage, and no industrial and agricultural distribution. Their health might be attributed to little anthropogenic impact on the sampling sites. The sites with a “very poor” and “poor” status were densely populated with a high degree of cluster, and certain agricultural activities around. Artificial activities had a big impact on these sites. Of all tributaries, rivers with an “excellent” and “good” condition accounted for 47.23%, while rivers with a “very poor” and “poor” status accounted for only 2.78%. In general, the health status of 36 important tributaries of TGR was generally in a good shape. The ecological condition of Guandu River and Daxi-F River2 was “excellent”, mainly because the areas through which the two rivers flowed were sparsely populated, with undeveloped industry and agriculture, and little the impact of anthropogenic activities on the rivers. The health status of Taohua River was “fair”, and relatively the poorest, mainly as it flows through a few populous villages and towns, and its basin was distributed with industrial and mining enterprises. Therefore, artificial activities generated a relatively big and complex impact on the river. In general, the health status of the rivers from the TGR dam to the upper reaches displayed a gradually deteriorating trend, consistent with the conclusions reached by Chi et al.^20^ on the ecological status of some tributaries of the TGR, probably due to a gradually increasing population, gradual development of industry and agriculture from the dam to the upper reaches, especially the position of the upper reaches in the main urban areas of Chongqing, where there were more human activities, resulting in a bigger impact on rivers. Six rivers, R03–R08, are located in Badong County, Hubei Province and Wushan County and Wuxi County, Chongqing Municipality. These three counties are less developed in terms of industry and agriculture, with a small population size in the river basin, little pollution discharged into rivers and less anthropogenic impact. Therefore, the health status of these six rivers is “excellent” or “good”. Four rivers, R28–R31, show a continuous “good” status, mainly due to undeveloped industry and agriculture, a small population size and little impact of human activities in the basin. Four rivers, R09–R12, exhibit a continuous “fair” status, mainly due to a large population living along the rivers, and possibly excessive amount of domestic pollution discharged into the rivers. Four rivers, R32–R35, located in the main urban areas of Chongqing, have a high urban land coverage rate, developed industry and agriculture, and a dense population^26^. Therefore, the health status of these four rivers is relatively poor due to the big anthropogenic impact. As to the results and analysis of the ecological status of sampling sites and rivers, the health status of rivers may be mainly related to urban land coverage, population concentration, industrial and agricultural distribution and human activities^7,9,27^. Although the health status of rivers is generally good, there is a significant difference between different sampling sites. The proportion of sampling sites with “very poor” and “poor” status is relatively high. The protection of river ecosystem does not look optimistic, and accordingly, protective efforts should be beefed up. Specifically, to strengthen the long-term monitoring of the health status of the TGR, regular follow-up assessments (e.g., with an interval of 3–5 years) of the health status of the reservoir and its tributaries should be conducted using the B-IBI constructed in this study. The obtained monitoring results are then used to guide the vegetation restoration, land use, town planning and distribution of industry and agriculture in the TGR area to reduce anthropogenic interference, prevent soil and water losses and curb the release of agricultural non-point source pollutants, domestic pollutants and industrial pollutants, thereby improving the health status of the TGR and its tributaries.

The screening criteria for reference sites and impaired sites in this study are feasible and workable. However, the screening criteria for reference sites and impaired sites only involve the anthropogenic disturbance, vegetation coverage, population distribution, and industrial and agricultural distribution around the sampling sites, without considering chemical properties of the water body at the sampling sites. Moreover, the number of reference sites is also small. Therefore, it is necessary to further study and perfect the selection criteria for reference sites and impaired sites, and identify the reference sites and impaired sites by combining the quantitative and qualitative methods^6,28^. In addition, although a number of indexes were used to assess the health status of the TGR and its tributaries in this study, the interactions among these indexes were not further analyzed. Analysis of the interactions among these indexes may be helpful to discover which species are more tolerant to pollutants in the investigated region.

In this study, we established B-IBI river health assessment to evaluate the health status of 36 important tributaries of TGR in China. This study is a transbasin and multi-river, comprehensive assessment which is characterized by complex basin types, involving a big study area and a number of rivers. Our research enriches the practice for B-IBI river health assessment and is beneficial for the improvement and promotion of B-IBI assessment method. The survey data and assessment results bear on the protection of water ecosystems of TGR and its important tributaries. In the meantime, they can provide some reference for assessing the health status of similar rivers around the globe.

## Methods

### Investigation method

**Figure 3:**
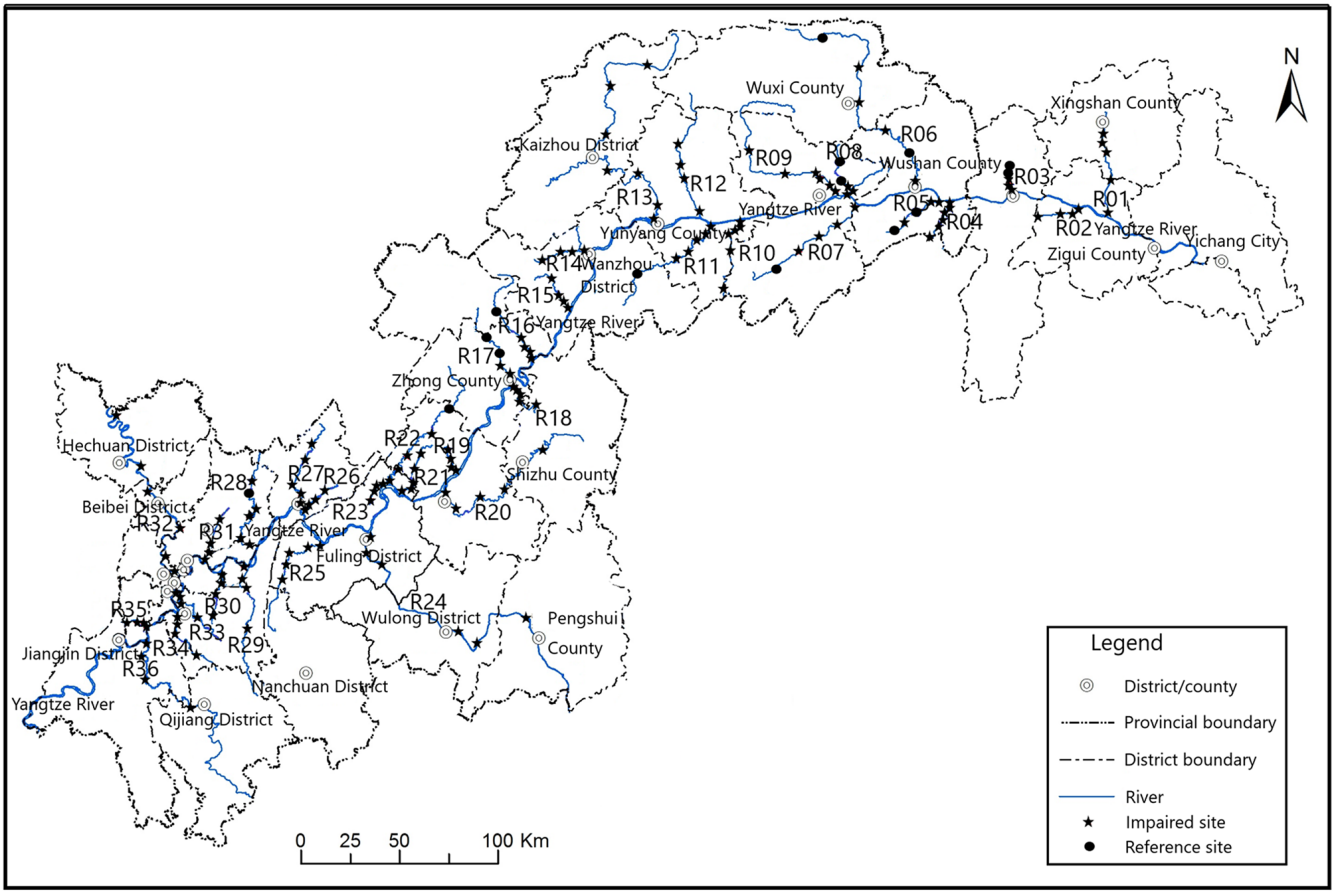
Schematic diagram of important tributaries of TGR and sampling points (plotted by ArcGIS 10.5, https://www.32r.com/soft/16101.html).

From March 2015 to December 2018, we surveyed 36 important tributaries of the TGR (Fig. 3) and conducted an investigation of macroinvertebrates. For the sake of convenience, we labeled tributaries from the reservoir dam to its tail area sequentially as R01–R36, i.e., R01 (Xiangxi River), R02 (Qinggan River), R03 (Shennong River), R04 (Baolong River), R05 (Guandu River), R06 (Daning River), R07 (Daxi-F River), R08 (Caotang River), R09 (Meixi River), R10 (Changtan River), R11 (Modao River), R12 (Tangxi River), R13 (Xiao River), R14 (Zhuxi River), R15 (Rangdu River), R16 (Ruxi River), R17 (Huangjin River), R18 (Dongxi River), R19 (Chixi River), R20 (Long River), R21 (Bixi River), R22 (Quxi River), R23 (Zhenxi River), R24 (Wu River), R25 (Lixiang River), R26 (Longxi River), R27 (Taohua River), R28 (Yulin River), R29 (Wubu River), R30 (Changtang River), R31 (Chaoyang River), R32 (Jialing River), R33 (Huaxi River), R34 (Yipin River), R35 (Daxi-J River), and R36 (Qi River).

This study was approved by the Environmental Protection Bureau of the Three Georges Reservoir.

A total of 175 sampling points were set up in all tributaries. Four parallel samples were taken from each sampling point. At least one sample was taken from each microhabitat (mainly including four microhabitats, i.e., shoal, deep pool, pebble and aquatic habitat). Parallel samples from the same sampling point were mixed together. The quantitative and qualitative sample collection methods were combined in this study. The quantitative collection was performed first, and then the qualitative collection for the same sampling point. The qualitative samples were collected by D-net. Quantitative samples of wadable sampling points were collected using a Surber net with an area of 0.3 m × 0.3 m. Quantitative samples of non-wadable sampling points were collected using a D-net with a bottom side length of 0.3 m. The collected samples were put into sample bottles (bags) and fixed with 5% formaldehyde solution. Then the samples were identified and classified under the laboratory conditions.

### Selection of reference sites and impaired sites

A reference site refers to a sampling point with no or little anthropogenic disturbance, while a impaired site refers to a sampling point subject to obvious anthropogenic disturbance^29^. A total of 15 reference sites and 160 impaired sites were selected from 175 sampling points based on anthropogenic disturbance, vegetation coverage, population distribution, and the distribution of industry and agriculture in the vicinity of the sampling site^6,8^ (Table 7, Supplementary Fig. 1).

**Table 7:** Assessment criteria for reference sites and impaired sites and the assessment outcomes.

Site type	Reference	Impaired
Anthropogenic disturbance	No or slight disturbance	Serious disturbance
Vegetation coverage	A high vegetation coverage with little agricultural vegetation	Serious vegetation damage with agricultural vegetation dominant
Inhabitant distribution	No	Yes
Distribution of industry and agriculture	No industry, and no or sporadically distributed agriculture	Yes
Number of sites (n)	15	160

### Creation and selection of the assessment metric index system

With reference to the river health assessment indexes in China^13,15,18,24^, North America^6,24^ and Europe^5^, and based on the ecological characteristics such as species composition and abundance, sensitivity, tolerance and functional feeding groups, we constructed 26 candidate metrics (Table 8) for B-IBI. These candidate metrics have significant or noticeable response to human activities, and normally, can be applied to relatively large geographic areas; therefore, they can be used to indicate the ecological quality of rivers^6,23,24^. Among these metrics, 17 were associated with species composition and abundance, which included the total number of taxon, the number of EPT taxa, the number of crustacean and mollusca taxa, the number of ephemerida taxa, the number of pteroptera taxa, the number of trichoptera taxa, the number of diptera taxa, the number of chironomidea taxa, the percentage of EPT, the percentage of crustacean and mollusca, the percentage of ephemerida, the percentage of pteroptera, the percentage of trichoptera, the percentage of dipteral, the percentage of chironomidea, the percentage of oligochaeta and the Shannon-Weiner diversity index. Species composition and abundance-related indexes reflect the diversity of macrobenthic communities. An increase in species diversity is associated with the improvement of community health, which indicates that the niche space and food sources are sufficient to support the survival and reproduction of multiple species. The candidate metrics related to sensitivity and tolerance in this study were the number of sensitive taxa, the number of tolerant taxa, the percentage of dominant species and the percentage of the top three dominant species. Different zoobenthos show different degrees of sensitivity and tolerance to the influencing factors in the river habitat, for which these characteristics can be used to assess the health status of the river. In addition, the taxa and percentage of functional feeding groups are closely associated with their living environment, and the parameters that were used to represent functional feeding in this study were the percentages of shredders, herbivores, filterers, scrapers and predators. Some of the representative images of the identified taxa were shown in Supplementary Fig. 1E,F.

**Table 8:** Candidate parameters for B-IBI and their response to anthropogenic disturbance.

Parameter attribute	Serial number	Biological parameter	Parameter description	Response to disturbance	References
Species composition and abundance	M1	Total taxa	Total taxon number of the benthic fauna in the sample	Decrease	Barbour et al.^23^
	M2	EPT taxa	Number of the ephemerida + trichoptera + Plecoptera taxa in the sample	Decrease	Barbour et al.^23^
	M3	Crustacean + Mollusca taxa	Number of the crustacean + mollusca taxa in the sample	Decrease	Qu et al.^24^
	M4	Ephemerida taxa	Number of the ephemerida taxa in the sample	Decrease	Barbour et al.^23^
	M5	Pteroptera taxa	Number of the pteroptera taxa in the sample	Decrease	Qu et al.^24^
	M6	Trichoptera taxa	Number of the trichoptera taxa in the sample	Decrease	Barbour et al.^23^
	M7	Diptera taxa	Number of the diptera taxa in the sample	Decrease	Barbour et al.^23^
	M8	Chironomidea taxa	Number of the chironomidea taxa in the sample	Decrease	Barbour et al.^23^
	M9	EPT (%)	Number of the EPT individuals/the total number of individuals in the sample	Decrease	Barbour et al.^23^
	M10	Crustacean + Mollusca (%)	Number of the crustacean and mollusca individuals/the total number of individuals in the sample	Decrease	Qu et al.^24^
	M11	Ephemerida (%)	Number of the ephemerida individuals/the total number of individuals in the sample	Decrease	Barbour et al.^23^
	M12	Pteroptera (%)	Number of the pteroptera individuals/the total number of individuals in the sample	Decrease	Qu et al.^24^
	M13	Trichoptera (%)	Number of the trichoptera individuals/the total number of individuals in the sample	Decrease	Barbour et al.^23^
	M14	Diptera (%)	Number of the diptera individuals/the total number of individuals in the sample	Increase	Barbour et al.^23^
	M15	Chironomidea (%)	Number of the chironomidea individuals/the total number of individuals in the sample	Increase	Barbour et al.^23^
	M16	Oligochaeta (%)	Number of the oligochaeta individuals/the total number of individuals in the sample	Increase	Barbour et al.^23^
	M17	Shannon–Weiner diversity index	$\left| {\text{r}} \right|$ where *H* is an diversity index, *n* is the total number of individuals, *S* is the number of taxa, and *n*_*i*_ is the number of the individuals of the *i*th taxon	Decrease	Qu et al.^24^
Sensitivity and tolerance	M18	Intolerant taxa (PTV ≤ 3)	Number of the taxa with a tolerance value ≤ 3	Decrease	Barbour et al.^23^
	M19	Tolerant taxa (PTV ≥ 7)	Number of the taxa with a tolerance value ≥ 7	Increase	Barbour et al.^23^
	M20	Dominant species (%)	Individual number of the most dominant species/the total number of individuals in the sample	Increase	Barbour et al.^23^
	M21	Top three dominant species (%)	Individual number of the top three dominant species/the total number of individuals in the sample	Increase	Qu et al.^24^
Functional feeding	M22	Shredders (%)	Number of the shredder individuals/the total number of individuals in the sample	Decrease	Qu et al.^24^
	M23	Herbivores (%)	Number of the herbivore individuals/the total number of individuals in the sample	Increase	Qu et al.^24^
	M24	Filterers (%)	Number of the filterer individuals/the total number of individuals in the sample	Increase	Qu et al.^24^
	M25	Scrapers (%)	Number of the scraper individuals/the total number of individuals in the sample	Decrease	Qu et al.^24^
	M26	Predators (%)	Number of the predator individuals/the total number of individuals in the sample	Decrease	Qu et al.^24^

The selection of core metrics for B-IBI mainly includes three steps: analysis of distribution range of candidate metrics, analysis of discriminant ability of candidate metrics and analysis of correlation between candidate metrics^23^.

### Analysis of distribution range of candidate metrics

According to the numerical value of each biological metric in the reference site, an initial analysis was conducted to exclude the following two types of metrics: metrics with excessive nought values, which did not meet the requirement for a universal applicability; metrics with a scatter value distribution, and a standard deviation greater than or equal to the mean, indicating that the standard deviation of this value was relatively big and unstable, thereby unsuitable to be used as biological metrics^6^.

### Analysis of discriminant ability of candidate metrics

After analyzing the distribution range of candidate metrics, those unsuitable for biological evaluation were eliminated. The distribution of the remaining eligible metrics for the reference site and the impaired site was analyzed using the box-plot, to mainly compare the distribution range of the 25th quantile to the 75th quantile of the reference site and the impaired site and the overlap of “box” InterQuartile Range (IQR), and judge which biological metrics could best distinguish between the reference site and impaired site. An IQ value ≥ 2 indicates a small overlapping part between the reference site and the impacted site, which means a significant difference in the related parameter between the reference site and the impacted site, suggesting a noticeable response to human activity^6,24^. The IQ scoring criteria were as follows^6,24^: 3 point, no overlapping between the two box bodies; 2 points, the box bodies have a small part of overlapping, but the median of neither body falls within the limits of its counterpart; 1 point, most parts of the box bodies overlap, and the median of at least one box body lies within the limits of its counterpart; 0 points, one box body falls within the limits of the other, or the medians of each body are within the other’s limits.

### Correlation analysis of candidate metrics

Pearson correlation analysis was further performed of the metrics that met the preliminary conditions. If the correlation coefficient $\left| {\text{r}} \right|$ between two metrics is greater than 0.75, and they are intrinsically linked. Then most of the information reflected is overlapping. Therefore, it is OK to select one of them. If no intrinsic connection is found between two metrics, then both metrics can be selected even if the correlation coefficient is greater than 0.75^8^.

After screening through the above three steps, core metrics of the B-IBI are finally determined.

### Construction of B-IBI

The core biological metrics screened out by the above method were used as the metrics for final biological assessment. The metrics used for biological assessment were standardized using the ratio scoring method, to unify the evaluation metric^23^.

1. For a metric that decreased with increasing interference, the metric was normalized by dividing the value of this metric at each sample point with the 95% quantile of all sample points:
  $${\text{V}}_{{\text{i}}}^{\prime } = {\text{V}}_{{\text{i}}} /{\text{V}}_{{{95}\% }} ;$$
2. For a metric that increased with increasing interference, the metric was normalized by using the 5% quantile of this metric at all sample points as the reference object:
  $${\text{V}}_{{\text{i}}}^{\prime } = \left( {{\text{V}}_{{{\text{MAX}}}} - {\text{V}}_{{\text{i}}} } \right)/\left( {{\text{V}}_{{{\text{MAX}}}} - {\text{V}}_{{{5}\% }} } \right),$$
  where V_i_^′^ is the normalized value of the metric at the ith sampling point; V_i_ the actual value of the metric at the ith sampling point; V_95%_ the 95% quantile of the metric; V_5%_ is the 5% quantile of the metric; V_MAX_ is the maximum value of this metric in all sampling points. The health thresholds of 5% quantile and 95% quantile can eliminate extreme abnormal values and retain most of biological information.

### B-IBI assessment criteria

The 95% quantile of B-IBI distribution of all the sections/tributaries used for the health threshold can eliminate extreme abnormal values and retain most biological information. The distribution range lower than this value is divided into four portions, and the quartile close to the 95% quantile indicates a small disturbance. The biological integrity grade and the corresponding range of IBI^6^ are determined according to the 95% quantile and the quartile value, and the section/river health was classified into five grades, namely, excellent, good, fair, poor and very poor.

## Supplementary information

Supplementary Legend.

Supplementary Figure 1.
