# Supplementary material for: Health assessment of important tributaries of Three Georges Reservoir based on the benthic index of biotic integrity
Source: Sci Rep. 2020 Oct 30;10:18743. doi: 10.1038/s41598-020-75746-7 (PMC7599234; doi:10.1038/s41598-020-75746-7)
Supplement: Supplementary file 1 — Supplementary Legend. [file 41598_2020_75746_MOESM1_ESM.docx]

Supplementary Figure 1. Representative images of the sample sites, the identified taxa, field sampling and indoor laboratory analysis. A, reference sites, Dawan, Daning River. B, reference sites, Zieba, Guandu River. C, impaired sites, Duihe, Dongxi River. D, impaired sites, Zhujiaqiao, Taohua River. E, *Bellamya aeruginose*. F, *Neoperla niponensis*. G, field sampling. H, Indoor laboratory analysis.
