# Supplementary figures and images for: Health assessment of important tributaries of Three Georges Reservoir based on the benthic index of biotic integrity
Source: Sci Rep. 2020 Oct 30;10:18743. doi: 10.1038/s41598-020-75746-7 (PMC7599234; doi:10.1038/s41598-020-75746-7)

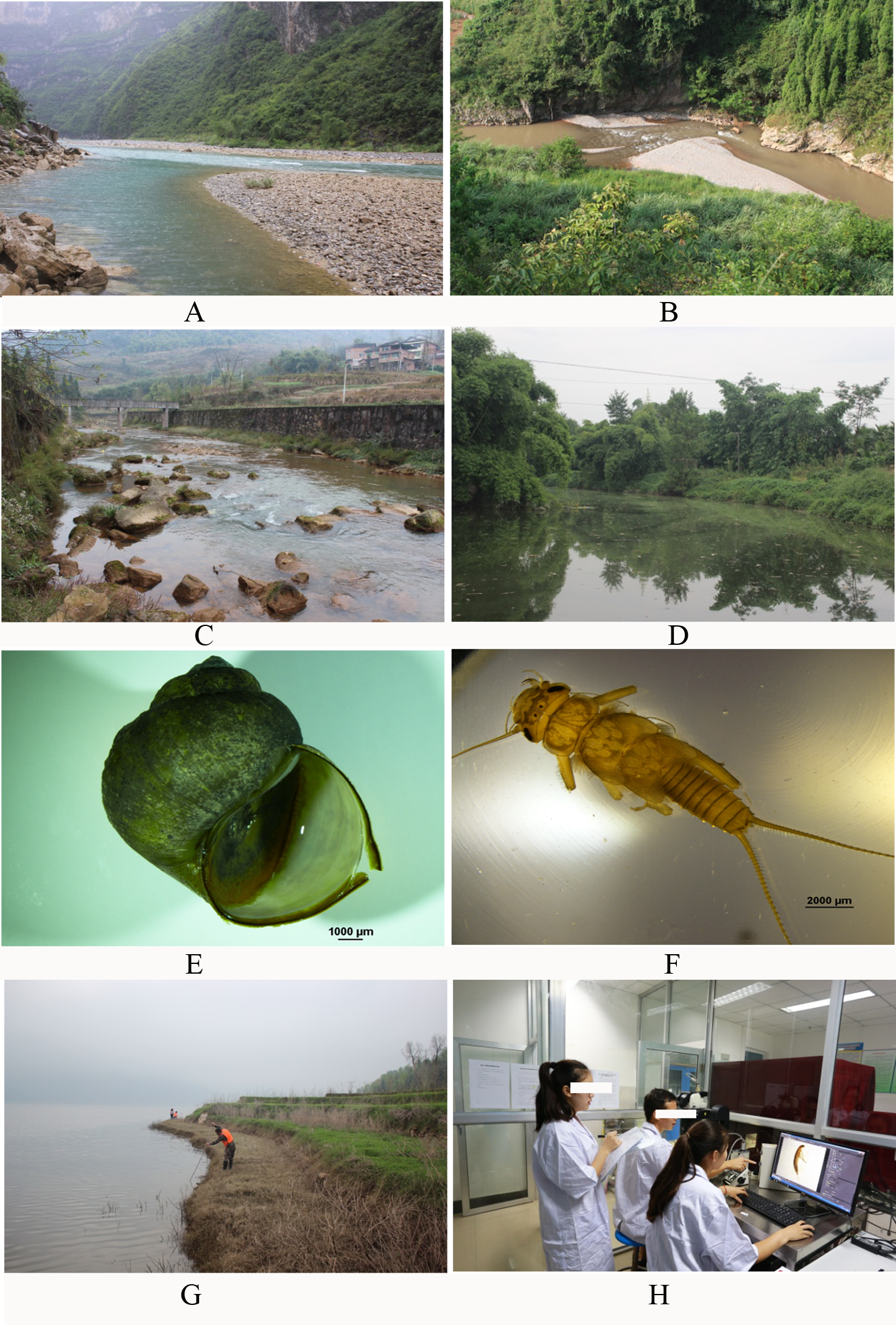

Supplement: Supplementary file 2 — Supplementary Figure 1. [file 41598_2020_75746_MOESM2_ESM.tif]
